# Spheroid Formation and Recovery Using Superhydrophobic Coating for Regenerative Purposes

**DOI:** 10.3390/pharmaceutics15092226

**Published:** 2023-08-29

**Authors:** María del Carmen Morán, Francesca Cirisano, Michele Ferrari

**Affiliations:** 1 Departament de Bioquímica i Fisiologia, Secció de Fisiologia—Facultat de Farmàcia i Ciències de l’Alimentació, Universitat de Barcelona, Avda. Joan XXIII, 27-31, 08028 Barcelona, Spain; 2 Institut de Nanociència i Nanotecnologia—IN2UB, Universitat de Barcelona, Avda. Diagonal, 645, 08028 Barcelona, Spain; 3 CNR-ICMATE Istituto di Chimica della Materia Condensata e di Tecnologie per l’Energia, Via De Marini, 6, 16149 Genova, Italy; francesca.cirisano@ge.icmate.cnr.it

**Keywords:** fibroblasts, keratinocytes, proliferation, recovery, regenerative, removal, spheroids, superhydrophobicity

## Abstract

Cell therapies commonly pursue tissue stimulation for regenerative purposes by replacing cell numbers or supplying for functional deficiencies. To this aim, monodispersed cells are usually transplanted for incorporation by local injection. The limitations of this strategy include poor success associated with cell death, insufficient retention, or cell damage due to shear forces associated with the injection. Spheroids have recently emerged as a model that mimics an in vivo environment with more representative cell-to-cell interactions and better intercellular communication. Nevertheless, cost-effective and lab friendly fabrication and effectively performed recovery are challenges that restrict the broad application of spheroids. In this work, glass surfaces were modified with an environmentally friendly superhydrophobic coating. The superhydrophobic surfaces were used for the 3D spheroid preparation of fibroblasts (3T3 cell line) and keratinocytes (HaCaT cell line). The effectiveness of the spheroids to be recovered and grown under 2D culture conditions was evaluated. The morphology of the migrated cells from the 3D spheroids was characterized at the nano-microscale through 3D profilometry. The results demonstrated improved adhesion and proliferation in the migrated cells, both advanced properties for regenerative applications.

## 1. Introduction

Cell culturing is the answer to many crucial questions regarding both basic science and translational research. The most remarkable properties of using cell lines include the homogeneity and reproducibility of the generated data. In vitro cell cultures have been widely used to assess cell biology, drug action, mechanisms of diseases, and tissue-engineering development, among others [1,2].

From a therapeutical point of view, cell therapy is a useful option to aid recovery from traumatic damage, inefficient wound repair, degenerative diseases, or cancer. Cell therapies commonly pursue the stimulation of tissue for regenerative purposes or replacing cell numbers or supplying for functional deficiencies. For example, erythrocyte transfusions treat anemia [3], bone marrow transplants replace diseased marrow and regenerate hematopoietic cell lineages [4], and chondrocytes are applied by injection to treat injured full thickness cartilage [5]. For these purposes, autologous sources (i.e., the patient’s own cells) in the form of monodispersed cells are usually transplanted for incorporation by local injection. The limitations of this strategy include the poor success associated with cell death, insufficient retention at the site or cell damage due to shear forces associated with the injection [6]. Cell–cell and cell–matrix signaling limitations are responsible for losing viability due to exposure to a harsh and uncontrolled microenvironment [7,8,9]. Moreover, the data from such a cellular system do not mimic the complex cellular interactions between different cell types and the extracellular matrix (ECM) of an in vivo environment [10].

To address this weakness, considerable research concerning the design of bioartificial matrices or platforms promoting cellular growth in their native 3D orientation is currently projected [11]. In that regard, it has recently been reported how hydrophobicity/superhydrophobicity modulates cell adhesion and viability [12,13,14]. Highly water- and oil-repellent materials, including superhydrophobic, oleophobic or amphiphobic materials could be used in biological applications. However, the literature only provides a limited number of reports dedicated to promoting the formation of 3D spheroids [15]. Several assemblies have been designed based on two main approaches. For example, the hanging-drop method is based on gravity to prepare one or more spheroids in a single drop. This method uses a surface where several drops are placed, remaining attached as it faces down. In contrast, spheroids can be grown located in a sitting-drop position in the absence of any tilt. The sitting-drop method includes several disadvantages, all of them having in common the poor wettability of the substrate.

As reported by the present authors, 3D spheroids have recently been promoted on surfaces of differing wettability, from hydrophilic to superhydrophobic [15,16]. Furthermore, both tumoral and non-tumoral cell lines have been grown on the prepared substrates in view of possible discrimination. The role of surface properties in differentiating cell-size populations as a function of growth dynamics has been shown [16].

Consequently, effective delivery systems regulating cell survival, behavior, and function are required to efficiently transplant cells to the target sites. Recently, many studies have demonstrated that 3D-cultured cells enhanced viability [17], differentiation [18] paracrine secretion [19] and tissue regeneration [20,21] compared to 2D-cultured cells [22,23]. Spheroids ensure the cell–cell and cell–matrix interactions of the in vivo 3D microenvironments, in which cells exhibit improved proliferation, differentiation, and cellular function compared to cells grown in 2D culture [15,24]. Therefore, 3D-spheroid cultured cells become good alternatives for cell transplantation with great potential for cell therapy and tissue engineering due to their enhanced therapeutic and regenerative capacity.

The increasing biomedical application of spheroids still presents challenges. Cost-effective and lab-friendly fabrication and an effectively performed recovery are challenges that restrict the broad application of spheroids. In this sense, obtaining uniformly sized spheroids is a key feature that affects the diffusion of oxygen and nutrients inside the spheroids and the internal organization of proliferative and necrotic cells. Moreover, although hanging- and sitting-drop platforms have demonstrated excellent results in preparing 3D spheroids using both cell lines and patient cells, the effective retrieval of 3D spheroids is a bottleneck for further applications, especially for closed microfluidic or hanging-drop systems. However, it has recently been shown that using a sitting-drop strategy where the surface has been conveniently modified can assist in recovery from generated 3D spheroids [25]. Finally, although there have been years of preclinical research, there is a lack of standard imaging and analytical protocols. Advanced imaging and analysis tools for in situ spheroid preparation and evaluation are needed [26].

Given its potential applications in cell-based therapy, this study aimed to verify the 3D spheroid preparation on highly hydro-repellent surfaces for the viability, growth, morphology, and proliferation capacity of the derived cells. In this work, the preparation and physical characterization of highly water-repellent surfaces to develop and characterize 3D spheroids derived from two skin representative cell lines, such as fibroblasts (3T3 cell line) and keratinocytes (HaCaT cell line), was carried out. The effectiveness of the obtained spheroids in being recovered and transferred to make possible the growth of the derived cells under a 2D monolayer culture as a measure of cell viability was performed. The morphology characterization of the derived cells after spheroid formation and recovery was evaluated at the nano-microscale through advanced techniques, such as 3D profilometry.

## 2. Materials and Methods

### 2.1. Materials

The superhydrophobic surfaces were prepared by dispersing fumed silica nanoparticles (EVONIK HDK H15) purchased from Degussa (Hannover, Germany), with primary particles of about 5–30 nm in size, in a commercial fluoropolymer blend, a fluorosilane polymer (0.1 wt.%) in a hydrofluoroether solvent.

High glucose DMEM medium (4.5 g/L glucose), L-glutamine solution (200 mM), fetal bovine serum (FBS), and penicillin–streptomycin solution (10,000 U/mL penicillin and 10 mg/mL streptomycin), were purchased from Lonza (Verviers, Belgium). In addition, trypsin–EDTA solution (170,000 U/L trypsin and 0.2 g/L EDTA) and phosphate-buffered saline (PBS) were also purchased from Lonza. The 75 cm^2^ flasks and the culture dishes were obtained from TPP (Trasadingen, Switzerland).

### 2.2. Methods

#### 2.2.1. Surface Preparation and Characterization

A glass substrate was spray-coated employing 2.0 g/L of the above dispersion [27]. The resulting SHS were cleaned with deionized water to remove any physical adsorbed impurities potentially affecting cell viability and to assess in situ their high hydrophobicity.

Surface wettability was investigated by contact angle (CA) measured by ASTRA view drop-shape tensiometer (developed at CNR-ICMATE [28]) at room temperature using MilliQ high purity grade water as standard (Milli-Pore, Burlington, MA, USA) and an ion-exchange microfiltration system. Drops of about 5 μL were gently placed onto the surface, and contact angle was monitored up to spreading equilibrium. Moreover, 3D confocal and interferometric profilometry (Sensofar S-NEOX, Terrassa, Spain) was used to evaluate the surface roughness and structure of SH samples as confocal images. To allow a large scanned-surface, 3D profilometry was chosen for its ease and fast non-destructive use. This surface characterization was carried out according to the standard ISO 25178.

#### 2.2.2. Cell Cultures

Murine Swiss albino fibroblasts (3T3 cell line) and immortal human keratinocytes (HaCaT cell line) were grown in high-glucose DMEM medium supplemented with 10% (*v*/*v*) FBS, 1% (*v*/*v*) L-glutamine and 1% (*v*/*v*) antibiotic mixture at 37 °C and 5% CO_2_. Cells were cultured in 75 cm^2^ culture flasks and routinely split when cells were approximately 80% confluent.

#### 2.2.3. Cell Culture in Superhydrophobic Substrates

The small volume required for seeding the cells was confined into delimited areas of the coated samples fixed by a Viton (fluorinated elastomers) O-ring because of the SH repellence of the medium culture, to avoid the sample floating in the Petri dish.

Cells were seeded at two different cell densities (2 × 10^5^ cells/ mL and 2 × 10^6^ cells/mL). Cells were incubated for 48 h under standard conditions. Optical microscopy through a Nikon inverted microscope equipped with a video camera (Moticam 1080 HDMI and USB) was used to monitor in situ the cell morphology and growth. Images were analyzed with the image processor Motic Images 3.0 software, Motic (Xiamen, China).

#### 2.2.4. Spheroid Recovery and Growth under Standard 2D Conditions

For spheroid recovery, the medium containing the spheroids was gentle aspirated and fully expelled three times before aspirating for delivery. The collected spheroids were dispersed in 24-well plates containing complete DMEM medium (see above) under standard conditions of temperature and CO_2_. Cell migration and growth evolution under standard monolayer conditions were checked over time.

#### 2.2.5. Profilometry Studies

Cells were seeded at the above densities onto the coated glasses in standard atmosphere, temperature, and time conditions. After elimination of the spent medium, cells were fixed with 4% (*v*/*v*) paraformaldehyde for 15 min. Fixed cells in sterile PBS and low temperature (approximately 5 °C) were maintained until the point to be scanned.

Confocal mode was used to analyze the entire surface areas containing cells delimited by O-rings. Cells on selected areas were chosen, and the corresponding profiles analyzed with the SensoSCAN software (Sensofar, Terrassa, Spain). Morphological parameters (surface factor, height, and volume) were compared with those derived from control cells (cells cultured under 2D standard and conditions).

#### 2.2.6. Statistical Analyses

Statistical analyses were performed using IBM SPSS Statistics 29 software (IBM, Armonk, NY, USA). One-way analysis of variance (ANOVA) was used to determine statistical differences between data sets, following the Scheffé post hoc tests for multiple comparisons. Differences were considered statistically significant at *p* < 0.001 (unless otherwise mentioned). Results are reported as means ± standard deviation. Significant differences are illustrated in the figures and tables with an asterisk or other superscript symbols.

## 3. Results

### 3.1. Surface Characterization

**Figure 1:**
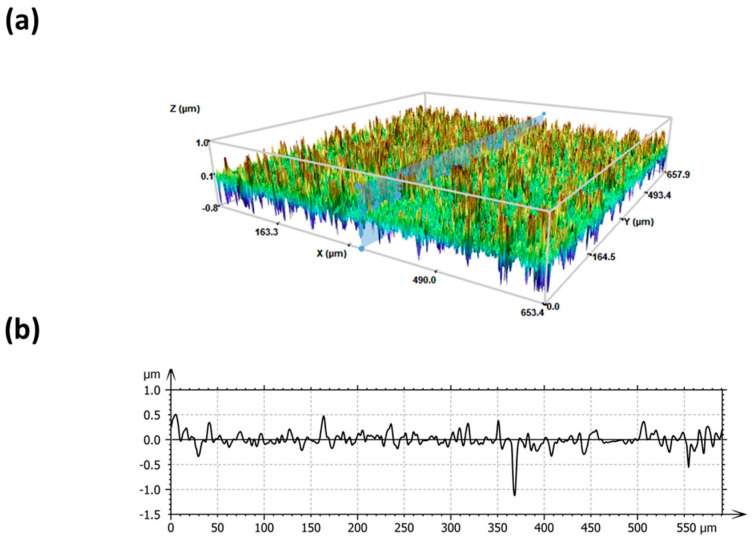
False color 3D view of SH surface (**a**) and a roughness profile taken from the surface by interferometric and confocal profilometer at 20× (**b**). The average roughness, Sa, is 180 nm.

The superhydrophobic surfaces (SHS) prepared by spray coating with a fluorinated polymers/silica nanoparticle dispersion were investigated, focusing on their wettability, morphology and roughness (Sa). The coated surfaces showed contact angles greater than 160° with hysteresis <5° evidenced by drops rolling off the surface. The topography, evaluated by 3D confocal and interferometric profilometry, was based on a double-scale nanometric roughness with an average value of 180 nm, conferring superhydrophobic properties. A representative 3D image at 20× and the related profile are shown in Figure 1.

### 3.2. Spheroid Preparation

**Figure 2:**
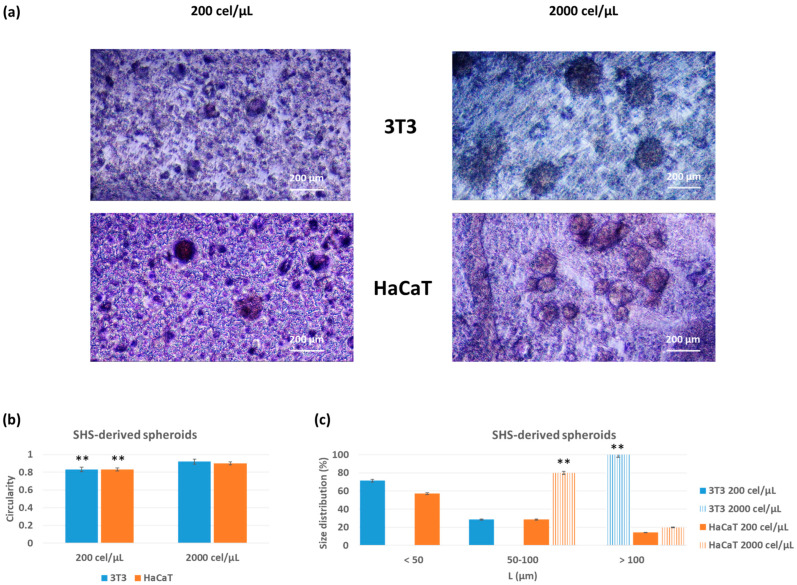
Phase contrast images (**a**), circularity (**b**) and size distribution (**c**) based on initial cell density (200 or 2000 cel/µL) after 48 h of incubation on highly water-repellent surfaces (SHS) for 3T3 fibroblasts and HaCaT keratinocytes. The results are reported as the average of more than 10 individual spheroids ± standard deviation. The scale bar represents 200 μm. ** (*p* < 0.001) indicates significant differences between initial cell density vales for the same cell line.

The ability of the superhydrophobic surfaces to generate spheroid-like structures was tested at two initial cell densities (200 and 2000 cell/μL) for an incubation time of 48 h. Of note, these surfaces are highly transparent, which allows the direct observation of cell aggregates using optical microscopy. The results demonstrated that the characteristics of the formed spheroids were highly dependent on the cell type and concentration (Figure 2).

The circularity of cell aggregates, as a measure of the effectiveness of 3D spheroid formation, was higher for systems formed at a higher cell density. Circularity values ranging from 0.83 to 0.92 for 3T3, and 0.83 and 0.90 for HaCaT, for initial cell densities of 200 and 2000 cel/μL, respectively, with significant differences (*p* < 0.001) between cell density values, for the same cell line. As a general trend, the increase in cell density favored the formation of larger 3D aggregates. In the case of the 3T3 fibroblasts, the size values of the generated aggregates (expressed in one dimension when considering their high circularity) ranged between 72% for aggregates <50 μm and 28% for aggregates in the range of 50–100 μm, and 100% for aggregates >100 μm, for initial cell densities of 200 and 2000 cel/μL, respectively.

In the case of the HaCaT keratinocytes, at the lower cell density, the size values of the aggregates ranged between 57% for aggregates <50 μm and 29% for aggregates in the range of 50–100 μm and 14% > 100 μm. By increasing 10 times the initial cell density, the size distribution was 80% for aggregates in the range 50–100 μm and 20% > 100 μm. Significant differences (*p* < 0.001) between cell density values were found in all cases.

### 3.3. Spheroid Recovery

**Figure 3:**
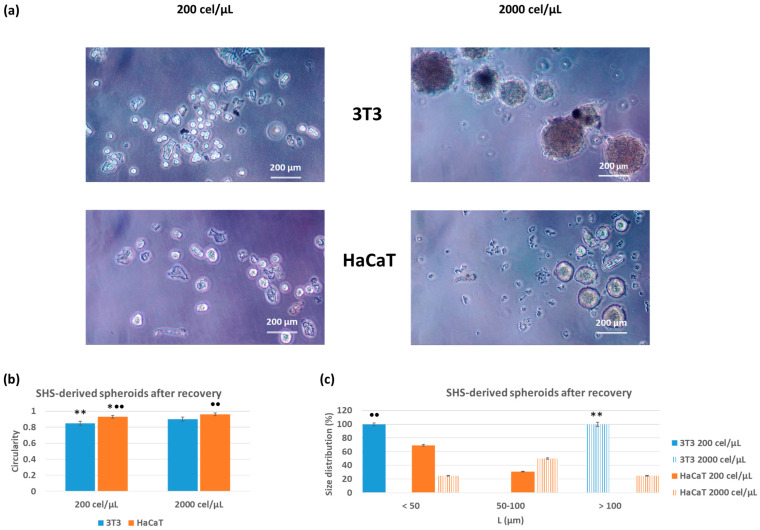
Phase contrast images (**a**), circularity (**b**) and size distribution (**c**) based on initial cell density (200 or 2000 cel/µL) of SHS-derived spheroids after recovery, for 3T3 fibroblasts and HaCaT keratinocytes. The results are reported as the average of more than 10 individual spheroids ± standard deviation. The scale bar represents 200 μm. * (*p* < 0.05) and ** (*p* < 0.001) indicate significant differences between initial cell density vales for the same cell line. ●● (*p* < 0.001) indicates significant differences between values before and after the recovery process.

Once the effectiveness in the formation of 3D spheroids was demonstrated, it was essential to evaluate their recovery from superhydrophobic surfaces. The high contact angle of the culture medium on these surfaces suggested that the medium containing the 3D spheroids could be easily isolated from surfaces under mild handling conditions. It was verified that simple suction through a micropipette tip allowed the isolation of the drop and the release into a new culture medium demonstrated no significant alteration of its characteristics (Figure 3). Thus, no noticeable differences were observed in terms of circularity, with values between 0.85 and 0.90 for 3T3, and 0.83 and 0.90 for HaCaT, for initial cell densities of 200 and 2000 cel/μL, respectively. In a similar way to that observed previously to the recovery step, significant differences (*p* < 0.001 3T3 and *p* < 0.005 HaCaT) were found as a function of the cell density values for the same cell line. The obtained results demonstrated a slight increase in circularity upon recovery, in all cases. The statistical analyses revealed no significant differences (*p* < 0.001) with respect to the circularity values before and after the recovery for the 3T3 cell line. When the HaCaT cell line was considered, however, these differences became significant (*p* < 0.001), but in a positive way. Even though the superhydrophobic surfaces (SHS) promoted a minimum contact angle, the circularity of the cellular aggregates in bulk conditions was be improved, as expected.

Concerning the size distribution after the recovery process, the obtained values suggested that the recovery could promote the formation of cell aggregates with lower dimensions, due to an increase in the percentage of aggregates in the range of <50 µm. However, this behavior depends on the cell line and cell density values. When 3T3-derived spheroids were considered, the size distribution after the recovery showed 100% for aggregates <50 μm (72% before the recovery) and 100% for aggregates >100 μm (identical % before the recovery), for initial cell densities of 200 and 2000 cel/μL, respectively. The statistical analyses demonstrated significant differences (*p* < 0.001) in comparison with cell aggregates before and after the recovery for 3D spheroids formed at the lower cell density. In a similar way to those found during formation, significant differences (*p* < 0.001) were found for 3T3-derived spheroids at the two different cell densities.

HaCaT keratinocytes seemed to be more sensitive to the manipulation, with a greater distribution of sizes after recovery. For the initial cell density of 200 cel/μL, the size distribution was 70% for aggregates <50 μm and 30% for aggregates in the 50–100 μm range. By increasing the cell density, the size distribution was 25% for aggregates <50 μm, 50% in the 50–100 μm range and 25% > 100 μm. However, no significant differences (*p* < 0.001) in comparison with cell aggregates before and after the recovery were found for spheroids formed at the two cell densities. No significant differences (*p* < 0.001) were found for recovered spheroids at the two cell densities.

Although these results have demonstrated that the recovery process did not induce dramatic modifications, the absence of statistical significance in size distribution after and before the recovery, especially in the case of spheroids formed at the lower cell density, might be related to the cells involved. In this sense, it is known that tissue-resident fibroblasts are mesenchymal cells that possess impressive plasticity in their ability to modify their properties according to the requirements the microenvironment [29]. This characteristic could explain changes in the size distribution of the 3T3-derived spheroids during recovery. On the other hand, the mutations in both alleles of p53 in HaCaT cells suggest a mechanism for their immortalization and inherent phenotype [30], resulting in less sensitivity to possible changes induced during recovery.

### 3.4. Cell Migration and Growth under 2D Conditions

**Figure 4:**
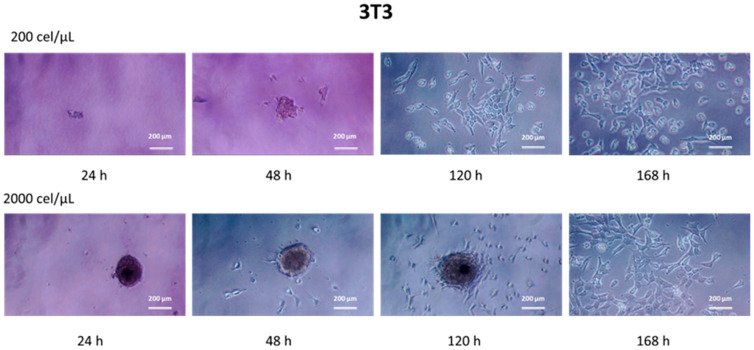

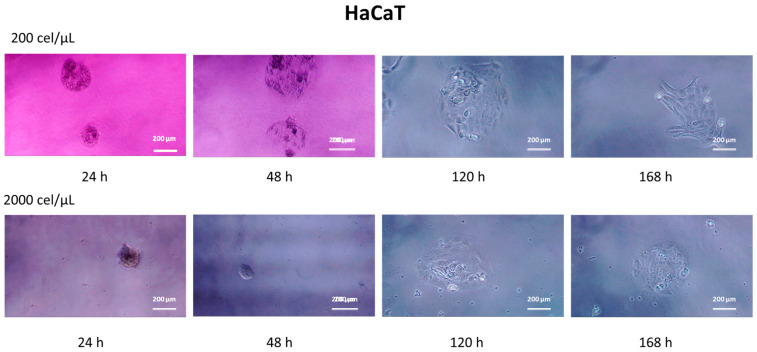
Phase contrast images of the temporal evolution of cells derived from spheroids formed in SHS depending on the initial cell density (200 or 2000 cel/µL) for 3T3 fibroblasts and HaCaT keratinocytes. The scale bar represents 200 μm.

The migration capacity from the cell aggregate, adhesion, and growth under standard culture conditions was evaluated once the ability of these spheroids to be recovered and transferred without significant modification in their characteristics was confirmed. Figure 4 shows representative images of this study based on the cell line, initial cell density and incubation time after recovery.

The results showed how the 3D spheroids remained stable in the cell culture conditions during the first 24 h from their transference. It was possible to visualize cell aggregates compacted depending on the cell line and initial cell density. By increasing the time (48 h), it could be observed how, in general, cells migrated from the cell aggregate. This behavior was highly dependent on the cell line and cell density. Thus, in the case of the 3T3 cell line (Figure 4), individual cells showing elongated shapes with typical bipolar or multipolar structures [31] migrating from the spheroid were visible. The number of migrated cells increased exponentially with time in a way almost independent of the initial cell density (Supplementary Materials, Figure S1a). Concerning the migrated distance, total migration from the spheroid core was achieved at 120 or 168 h after the recovery for initial cell densities of 200 or 2000 cel/µL, respectively.

Concerning the HaCaT cell line, the spheroids seemed to lose the compact structure and appeared with a more diffuse morphology as a consequence of their mode of growth in discrete patches [32]. The time increase gave way to cell patches compatible with standard epithelial cell growth. Diameters ranging up to 2.5 and 3.0 times the dimensions of the spheroids at 24 h after the recovery were found for 200 and 2000 cel/µL, respectively (Supplementary Materials, Figure S1b).

### 3.5. Morphological Characterization of Migrated Cells from the SHS-Derived Spheroids

Further investigations into the morphology of the migrated cells were performed once the viability and migration capability of the cells from the recovered spheroids was verified. For this purpose, 3D optical profilometry was used. Previous work in our lab demonstrated that this technique can be regarded as an interesting tool for observing changes in cell morphology during the screening of potential drugs and materials [33]. Furthermore, a key advantage of this technique is that it can be used in the absence of any fluorescent proteins or optically active dyes, and without the spatial limitation (cm^2^) of other techniques [34,35].

**Figure 5:**
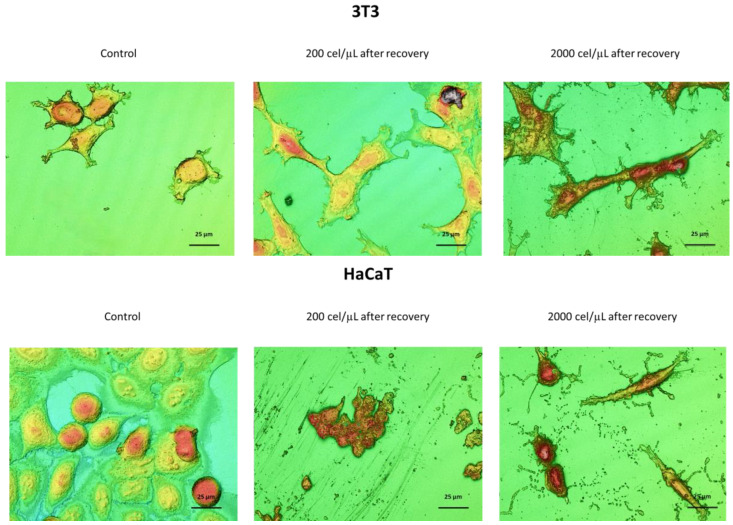
3D optical profilometry images in confocal mode (100× magnification) for 3T3 and HaCaT spheroids formed on SHS surfaces depending on the initial cell density (200 or 2000 cel/µL) for 168 h after recovery, and compared to control cells.

In this work, 3D profilometry was used to evaluate, qualitatively and quantitatively with nanometric resolution, the possible changes in the morphology of the cells transferred from the SHS-derived spheroids compared to control cells, that is, cells grown under standard 2D monolayer conditions (Figure 5). At a qualitative level, this study showed how the migrated cells showed slightly longer morphologies than those derived from a conventional 2D monolayer culture. These results suggest that the adhesion of these cellular entities is favored [36].

Interestingly, the analysis of the profiles allowed us to evaluate numerical changes in the dimensions of the derived cells compared to the control cells (Table 1). The profiles of the 3T3 fibroblasts under control conditions provided surface factor values around 1.7, compatible with their bipolar structure. However, after recovery from the 3D spheroids, this surface value increased to 2.6 and 3.2 for 200 and 2000 cel/µL, respectively. In the case of the HaCaT keratinocytes, the profiles obtained for control cells corroborated the spherical structure of these cells, showing surface values close to 1.0. The effect on 3D spheroid formation and recovery was demonstrated to be a consequence of the initial cell density, with surface factor values ranging between 1.3 and 7.0, for 200 and 2000 cel/µL, respectively.

In addition, the profile analyses of height demonstrated changes that were a function of the cell line and initial cell density. The height values of control cells resulted in values ranging from 1.6 to 1.9 µm for 3T3 and HaCaT, respectively. The effect of 3D spheroid formation and recovery produced opposite results at the lower cell density, with height values of 0.9 and 3.4 µm, for fibroblasts and keratinocytes, respectively. The increase of 10 times the initial cell density promoted a significant height increase of up to 4.1 and 4.3 µm for 3T3 and HaCaT, respectively.

Moreover, the 3D profiles allowed us to estimate the cellular volumes under the assayed conditions. Cells grown under control conditions showed cell volumes around 400 µm^3^ for both cell lines. The cell volume of the migrated cells from spheroids prepared at the lower initial cell density (200 cel/µL) demonstrated a slight decrease, which could be compatible with the manner of stress. However, by increasing the initial cell density during spheroid formation (2000 cel/ µL), the resulting cells revealed a significant increase in cell volume with values higher than 2.5 times the corresponding cell volume under control conditions.

As can be seen in Table 1, all these parameters (surface factor, height and volume values) demonstrated significant differences (*p* < 0.05 or *p* < 0.001) in comparison with control cells for the same cell line. Significant differences (*p* < 0.001) between cells at different initial cell densities for the same cell line were also found.

## 4. Discussion

In recent years, significant progress has been made in developing in vitro three-dimensional cell cultures to be used as models for in vivo tissue environments. Spheroids are cell aggregates in which the cell–cell and the cell–extracellular matrix contacts dominate [37,38]. As a result, it was shown that 3D-cell cultures exhibited increased levels of tissue-specific markers, retrieved tissue-specific functions, and developed various gene expression profiles compared to 2D-cultured cells [39]. Superhydrophobicity has been recently used to develop spheroids. However, most examples in the literature involve highly technological solutions [15,40,41]. In particular, the spray coating method employed in this work to prepare the superhydrophobic surfaces used to grow spheroids, has many advantages such as the possibility to use any type of substrate material by modifying its surface properties and its cost-effectiveness and ease of use. Under this approach, glass surfaces with high transparency, high superhydrophobic behavior and controlled roughness were prepared (Figure 1).

The high contact angle of the culture medium on these surfaces was demonstrated to assist the preparation of 3D spheroids of 3T3 fibroblasts and HaCaT keratinocytes after 48 h of incubation. Two initial cell densities (200 and 2000 cel/mL) were assayed, and their effect on the final properties of the generated spheroids was evaluated.

The superhydrophobicity also enabled the easy isolation of the spheroids from these surfaces under mild handling conditions without altering their physical characteristics, as demonstrated by the circularity values and size distribution before and after the recovery (Figure 2 and Figure 3, respectively). We used the circularity to characterize these spheroids before and after the recovery because this parameter is independent of the number of cells forming cell aggregates. As a general trend, the increase in cell density favored the formation of larger 3D aggregates. Concerning the recovery of 3D spheroids, this step is usually challenging for closed microfluidic or hanging-drop systems. Moreover, when spheroids are prepared on hydrogels, the recovery often involves chemical or enzymatic-mediated hydrogel disruption [42,43,44,45,46]. In these circumstances, without any hydrogel maintaining the cell aggregates together, in the absence of sufficient physical interaction between cells, the aggregates disassemble, and the physical compartmentalization disappears. In this work, however, adequate formation, i.e., spheroids with unaltered physical properties, enabled the process of sample recovery as well as the transference performed by simple pipetting.

The accurate characterization of 3D spheroids may be a weak point because most available techniques and protocols were initially designed for 2D culturing. Almost all the applied methods are colorimetric assays as indirect methods for determining viable cells [15]. Previous studies have demonstrated the homogeneous distribution of viable cells in superhydrophobic-induced spheroids [16] using the acridine orange/ethidium bromide (AO/EB) double staining. In this work, however, the recovered 3D spheroids were grown in conventional 2D (monolayer) conditions as a measure of cell viability and migration of the cells forming cell aggregates (Figure 4). The results demonstrated that cells remained alive with migrating capabilities over time, which are a function of both the cell line and the initial cell density. While the evolution of 3D spheroid formation has been widely studied over time, studies screening cell migration from the core spheroids are focused on tumoral cells as a measurement of tumor invasion [47].

In addition, in this work, the morphological characteristics of the migrated cells were deeply characterized at the nano-microscale through 3D profilometry (Figure 5 and Table 1). This approach represents a novelty compared to other studies in two different aspects. Firstly, to the author’s knowledge, there is no evidence of the morphology characterization of cells forming 3D spheroids. Secondly, 3D profilometry has become a non-invasive technique to be applied in life sciences [48]. In an approach developed for the authors of the present work, it was demonstrated how 3D morphological surface analysis matches standard biochemical methods to distinguish cells under control conditions and those derived upon incubation with precursors of proliferation and cell death.

An accurate characterization involves quantitative and qualitative analysis of the derived structures. The analysis at the nano-microscale through 3D profilometry demonstrated, at both the qualitative (elongated shape) and the quantitative (increased surface factor) levels, the increased adhesive properties of the derived cells. Moreover, the height and final volume increase demonstrated features compatible with cell proliferation procedures. This performance was observed in the two different cell lines, dependent on the initial cell densities in both cases. These results are in the light of cell adhesive behaviors on superhydrophobic surfaces where constant contact between cells is necessary to ensure cell division and proliferation on these surfaces [49]. Although cells cultured for short times on superhydrophobic surfaces hardly proliferate, cells adhering to the superhydrophobic surface over time produce an extracellular matrix (ECM) such as collagen, creating conditions suitable for cell proliferation. The results found in this work suggested that cells on 3D spheroids ensure the cell contact between them, resulting in cells with enhanced cell proliferation, especially for the migrated cells of SHS-derived 3D spheroids at the highest initial cell density. These results demonstrated that the migrated cells from SHS-based spheroids exhibited morphological changes compatible with improved adhesion and proliferation. Ongoing research is focused on 3D spheroid formation and characterization involving immune cells as tools for developing immunotherapy treatments.

## 5. Conclusions

In this work, superhydrophobicity was used to prepare 3D spheroids from representative skin cell lines such as fibroblasts and keratinocytes. The high transparency of the resulting superhydrophobic surfaces made the characterization in terms of circulatory and size distribution possible without any manipulation or recovery. In addition, in view of their therapeutical applications, the 3D spheroids recovery was performed under mild conditions without alteration to their physical properties. The cell viability of the 3D spheroids was demonstrated by their growth under 2D conditions. The migrated cells demonstrated increased cell attachment and proliferation, as shown by 3D profilometry analysis. The study demonstrated that superhydrophobicity can be effectively used as a platform for 3D spheroid formation and recovery, and for promoting added value to the biological characteristics in its application for regenerative purposes.

## Figures and Tables

**Table 1:** Morphological phenotype of cells cultured on various substrates. Surface factor (major axis length/minor axis length ratio), height and volume obtained from the corresponding cell profiles from 3T3 and HaCaT spheroids formed on SHS surfaces depending on the initial cell density (200 or 2000 cel/µL) for 168 h after recovery and compared to control cells. Results are reported as the average of ten independent cells ± standard deviation.

Sample	Surface Factor	Height (µm)	Volume (µm^3^)
Control	1.72 ± 0.50	1.59 ± 0.11	446.25 ± 68.25
3T3 200 cel/µL	2.61 ± 0.64 **	0.93 ± 0.26 **	300 ± 49.44 **
2000 cel/µL	3.24 ± 0.18 **^●●^	4.1 ± 0.63 **^●●^	1245.70 ± 163.68 **^●●^
Control	1.05 ± 0.04	1.91± 0.08	392.40 ± 86.26
HaCaT 200 cel/µL	1.31 ± 0.14 *	3.39 ± 1.37 **	315.40 ± 112.85 **
2000 cel/µL	6.98 ± 0.06 **^●●^	4.29 ± 0.64 **^●●^	956.70 ± 257.81 **^●●^

* (*p* < 0.05) and ** (*p* < 0.001) indicate significant differences between control cells for the same cell line. ^●●^ (*p* < 0.001) indicates significant differences between cells at different initial cell densities for the same cell line.

## Data Availability

The data presented in this study are available within this article.

## Supplementary Materials

The following supporting information can be downloaded at: https://www.mdpi.com/article/10.3390/pharmaceutics15092226/s1. Figure S1. Derived parameters of 3T3 fibroblasts (a) and HaCaT keratinocytes (b) migrated from 3D spheroids over time.
