# Supplementary material for: Spheroid Formation and Recovery Using Superhydrophobic Coating for Regenerative Purposes
Source: Pharmaceutics. 2023 Aug 29;15(9):2226. doi: 10.3390/pharmaceutics15092226 (PMC10538210; doi:10.3390/pharmaceutics15092226)
Supplement: Supplementary file 1 [file pharmaceutics-15-02226-s001.zip › pharmaceutics-2531256-supplementary.pdf]

(a)

(b)

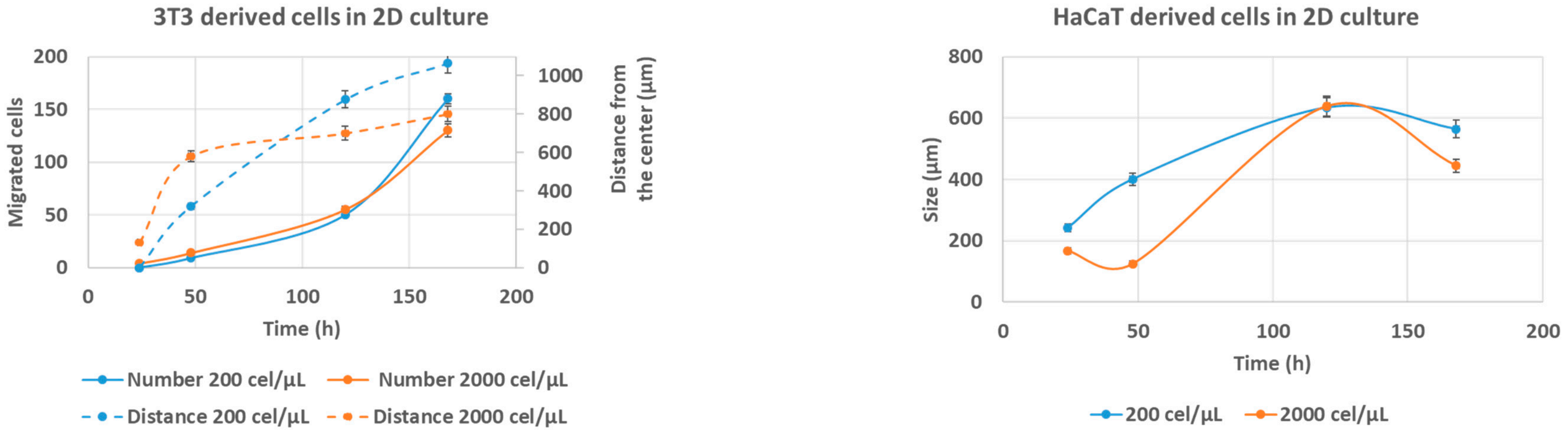

Figure S1. Derived parameters of 3T3 fibroblasts (a) and HaCaT keratinocytes (b) migrated from 3D spheroids over time.
